# Remission of Acute Food Protein-Induced Enterocolitis Syndrome Confirmed by Oral Food Challenges in Japan

**DOI:** 10.3390/nu14194158

**Published:** 2022-10-07

**Authors:** Koji Nishimura, Kiwako Yamamoto-Hanada, Miori Sato, Kenji Toyokuni, Hiroya Ogita, Tomoyuki Kiguchi, Yoshitsune Miyagi, Yusuke Inuzuka, Mayako Saito-Abe, Makoto Irahara, Fumi Ishikawa, Shigenori Kabashima, Yumiko Miyaji, Tatsuki Fukuie, Ichiro Nomura, Yukihiro Ohya

**Affiliations:** Allergy Center, National Center for Child Health and Development, 2-10-1 Okura, Setagaya-ku, Tokyo 157-8535, Japan

**Keywords:** allergens, dietary proteins, enterocolitis, food hypersensitivity, immune tolerance

## Abstract

The oral food challenge test (OFC) is the gold standard for evaluating the remission of food protein-induced enterocolitis syndrome (FPIES). Few acute FPIES remissions confirmed by OFC were reported. This study aimed to examine the OFC for Japanese children with acute FPIES to evaluate its remission. A retrospective cohort study was performed on children with acute FPIES with remission evaluation by OFC based on one food challenge dose (1/50, 1/10, 1/2, and full dose per day). Acute FPIES remission was observed in 65.2% of patients (15/23 patients). Vomiting episodes occurred with 1/50 full doses on the first day among 75% of positive patients. The median duration between the onset and OFC was 14 months (IQR, 8–24 months). Soy was the most common causative food, followed by egg yolk, milk, and wheat. All patients could receive OFC safely without intensive care unit care, based on the FPIES OFC protocol. The remission rate of acute FPIES was high. However, vomiting episodes commonly occurred with 1/50 full doses on the first day. This study suggested that our OFC protocol for acute FPIES was safe and feasible, but it might be safer for some patients to start at a minimal loading dose.

## 1. Introduction

Food protein-induced enterocolitis syndrome (FPIES) is a non-IgE-mediated food allergy with mild-to-severe shock reactions [1]. Acute FPIES is commonly present in infancy with repetitive emesis symptoms starting within 1–4 h after taking potential causal foods. This can be accompanied by lethargy, pallor, diarrhea, abdominal distention, and in its severe form, dehydration, hypotension, metabolic derangements, or shock. Acute FPIES is common and increasing in Japan and other countries [2,3,4].

The mechanism of FPIES is not well-understood. There is no laboratory test specific to FPIES. It is primarily clinically diagnosed, necessitating a thorough clinical history that reveals repeated reactions to the same food triggers with typical signs and symptoms, an improvement upon the removal of suspected triggers, and exclusion of other causes [5]. The oral food challenge test (OFC) is the gold standard procedure for diagnosing FPIES, but reactions to OFCs can be severe, with 15% presenting with hypotension and shock and 45–95% requiring treatment with intravenous fluids, steroids, or both in young children [6].

An OFC protocol for FPIES was established in the international consensus guidelines [1]. However, it has not yet been standardized in clinical practice or fully studied. In addition, the timing of OFC and its outcomes are unclear in evaluating remission of acute FPIES in patients already diagnosed with acute FPIES. This study aimed to examine OFCs for evaluating remission in Japanese children with acute FPIES.

## 2. Methods

### 2.1. Study Design, Setting, and Participants

This was a single-center, retrospective cohort study. Patient data were collected from medical records at the Allergy Center of National Center for Child Health and Development (NCCHD), the National Children’s Hospital in Tokyo, which is certified as one of the World Allergy Organization (WAO) Centers of Excellence. The inclusion criteria for this study were as follows: (1) acute FPIES diagnosed according to the criteria of the International Consensus Guidelines for the Diagnosis and Management of FPIES (Table 1) [1]; (2) having multiple episodes of acute FPIES; and (3) having OFC for FPIES remission evaluation from 1 January 2014 to 31 December 2019, at NCCHD. The definition of severity also conformed to the international consensus guidelines (1).

### 2.2. Variables and Data Source

We evaluated the patient’s age, sex, symptoms, age at onset of initial symptoms, age at diagnosis, causative foods, allergic disease comorbidities, perinatal history, family allergic disease history, laboratory and skin test results, and OFC-related information. The OFC data included the age, the dose of food tested, its outcomes, the timing of reaction, and treatments. All variables were derived from the patient’s electronic medical records at NCCHD. This study was approved by the Institutional Review Board of NCCHD (No. 2019-155). Oral informed parental consent was obtained for the use of patient data and storage as electronic medical records.

### 2.3. Laboratory Tests

We evaluated the following laboratory tests at the time of peripheral intravenous line placement before the OFC: levels of white blood cells, neutrophils, eosinophils, C-reactive proteins (CRPs), and thymus and activation-regulated chemokine [7]. The serum levels of total IgE and antigen-specific IgE were measured using an ImmunoCAP system (Thermo Fisher Scientific, Waltham, MA, USA). A contract laboratory company independently performed all laboratory tests.

### 2.4. Skin Prick Test

Skin prick test (SPT) was performed on the volar surface of the forearm with a bifurcated needle (Allergy Laboratories of Ohio, USA) and using commercial allergen extracts (Torii, Tokyo, Japan) or fresh food, along with positive (10 g/mL histamine) and negative controls (saline). We measured wheal sizes after 15 min. A wheal diameter equal to at least 50% of that obtained with the positive control was considered positive. An SPT response was scored as strongly positive if the wheal diameter was larger than 100% of that obtained with the positive control. SPT was conducted the day before the first day of OFC (see Supplementary Table S1 in this article’s Online Repository).

### 2.5. Oral Food Challenge Protocol

All patients underwent open OFC in our hospital for four days (see Supplementary Table S1 in this article’s Online Repository). In this approach, up-dosing was performed only once per day. This enabled us to monitor for delayed episodes of FPIES. A total challenge dose was set based on the severity of acute FPIES. A peripheral intravenous line was placed in all patients before the OFC. The final target challenge dose was set based on age severity, and the first started from a 1/50 dose of that. A challenge food dose was provided in a single portion a day, and the patient was observed for at least 4 h. If an IgE-mediated food allergy was suspected, the challenge food was administered in three divided doses every 40 min. If there were no symptoms caused by the 1/50 dose, it gradually increased over four days. A positive reaction to test foods was judged based on vomiting, diarrhea, or both symptoms. In moderate or severe reactions, the treatment was administered at the attending physician’s discretion; it included intravenous normal saline, intravenous corticosteroids, or both. A negative OFC on day 4 was defined as the remission of acute FPIES.

### 2.6. Statistical Analysis

All statistical analyses were performed with EZR (Saitama Medical Center, Jichi Medical University, Saitama, Japan) [8], which is a graphical user interface for R (The R Foundation for Statistical Computing, Vienna, Austria). A modified version of R commander was designed to add statistical functions frequently used in biostatistics. Nonparametric demographic and clinical data are reported as medians (interquartile range [IQR]), while parametric data are reported as means ± SD. Statistical analyses were performed using Fisher’s exact test, Mann–Whitney test, and Student’s *t*-test to investigate the background and clinical histories differences between OFC-positive and OFC-negative. The results were considered statistically significant if *p* values were less than 0.05.

## 3. Results

### 3.1. Patients and Clinical Features

**Figure 1:**
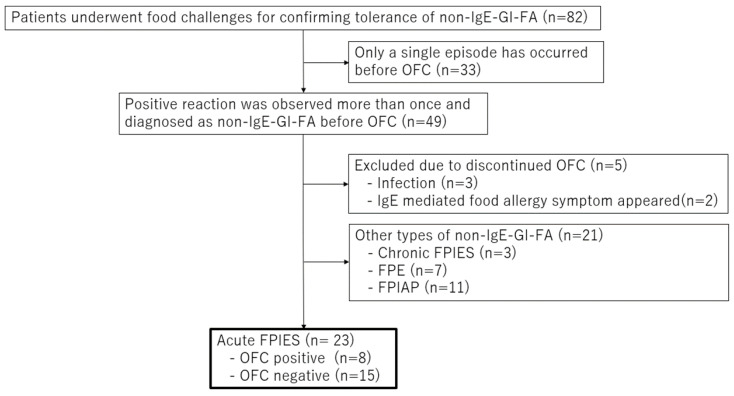
Flow diagram of the subject selection process. Non-IgE-GI-FA, non-IgE-mediated gastrointestinal food allergy; OFC, oral food challenge test; FPIES, food protein-induced enterocolitis syndrome; FPE, food protein-induced enteropathy; FPIAP, food protein-induced allergic proctocolitis.

**Figure 2:**
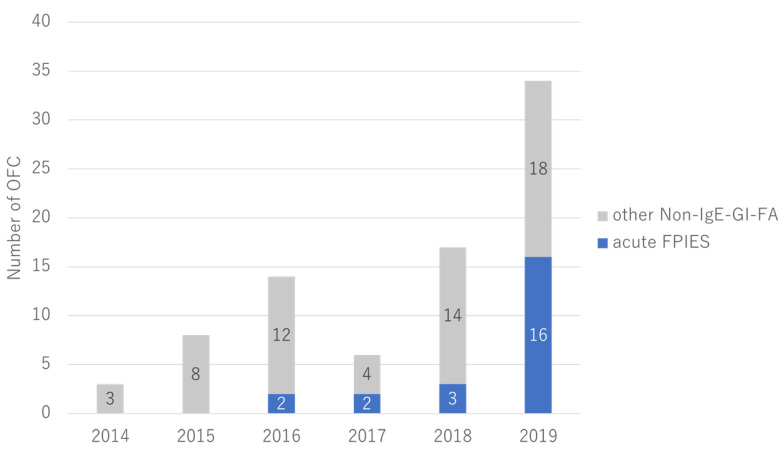
The distribution of follow-up OFC for acute FPIES and other non-IgE-GI-FA in our hospital in six years. Non-IgE-GI-FA, non-IgE-mediated gastrointestinal food allergies; OFC, oral food challenge.

**Figure 3:**
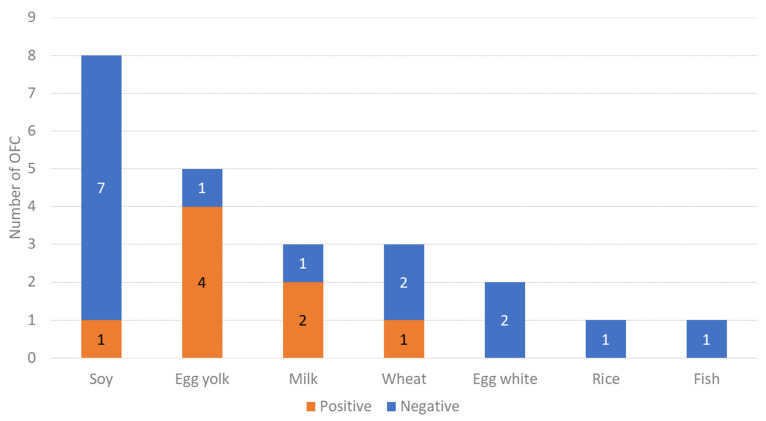
OFC outcomes for acute FPIES by causative food.

Twenty-three children with acute FPIES who had OFC for remission evaluation were included in this study (Figure 1). Positive and negative OFCs were observed in 8 and 15 patients, respectively. Acute FPIES remission was observed in 65.2% of patients (15/23 patients). The number of OFCs for acute FPIES remission evaluation increased annually (Figure 2). The patient’s characteristics were summarized in Table 2. The median ages at the onset of initial symptoms and diagnosis were 7.0 (interquartile range [IQR], 6.25–8.0) and 8.0 (IQR, 6.25–11.5) months, respectively. One patient took about five years to obtain an FPIES diagnosis. Causative foods (see Figure 3) were soy (*n* = 8), egg yolk (*n* = 5), milk (*n* = 3), wheat (*n* = 3), egg white (*n* = 2), rice (*n* = 1), and fish (*n* = 1). All participants reacted to a single food. IgE sensitization to causal food allergens was found in three (13%) patients. All patients who had SPT for causative foods (*n* = 22) were negative for SPT. When we did OFC for remission evaluation, the median age was 21 months (IQR, 15.3–32.0). The median duration between the onset and OFC was 14 months (IQR, 8–24). Patients with a negative OFC achieved FPIES remission at 13 months (median) after the onset and remained symptom free at home without food elimination. There was no significant difference in time from the onset of initial symptoms to follow-up OFC between OFC-positive and OFC-negative cases.

### 3.2. OFC Outcomes

Table 3 shows the clinical characteristic of patients with acute FPIES who had positive reactions in OFC. Adverse reactions due to OFC occurred in 35% of patients (8/23 patients). They all had vomiting, and 75% of vomiting episodes occurred with 1/50 serving size on the first day. Repeated vomiting was also commonly observed in seven out of eight patients. Pallor and hypotension did not occur during OFC. This study elucidated that acute FPIES remission was observed in 65.2% (n = 15) of infants (month of age, IQR, 14.5–31.5; range, 6–45) patients based on OFC (n = 23). In seven patients, reactions occurred within 4 h, but it was observed in one child after over 8 h. Egg yolk (n = 4) was the most common causative food in OFC-positive cases. Two (25%) had mild reactions among eight patients with positive reactions, and six (75%) had moderate reactions; no severe reactions were observed. Intravenous fluid (IVF) resuscitation and/or corticosteroids were administrated in four (50%) cases; none received any treatment in an intensive care unit (ICU).

## 4. Discussion

This study elucidated that acute FPIES remission was observed in 65.2% (n = 15) of infants (month of age, IQR, 15.3–32.0) patients based on OFC (n = 23). Vomiting episodes occurred with 1/50 full dose on the first day among 75% of positive patients. The median duration between the onset and OFC was 14 months (IQR, 8–24). Soy was the most common causative food in this study, followed by egg yolk, milk, and wheat. All patients could receive OFC safely without ICU care based on the FPIES OFC protocol. Our OFC protocol was feasible and practical.

As for remission, evaluation through OFC was commonly attempted within 12 to 18 months after the most recent reaction [1,9]. A study in Italy (n = 66) also demonstrated that 48% of patients with FPIES achieved tolerance at a mean age of 29 months (SD, 17 months) [10]. A study in Greece (n = 78) also performed OFC to evaluate their tolerance [11]. The time between the diagnosis and OFC (mean, 33 months; 95% CI, 27.1–38.9) was longer than that in our study, and 72.2% were negative for OFC. A longer elimination time might lead to higher remission rates for acute FPIES. Acute FPIES generally had a favorable prognosis; most patients had symptom resolution by 1–5 years of age [12]. However, a long elimination period imposes heavy burdens on children and their families. The sample size in this study was smaller than that in other studies. Further studies are required to determine the appropriate timing of OFC to confirm the remission of acute FPIES.

Although positive reactions occurred in 35% of study patients, there were no patients in the intensive care unit for further management for persistent or severe hypotension, shock, extreme lethargy, or respiratory distress. In our hospital, we planned the OFC by considering nutrition status and developmental catch-up after having long intervals from the initial diagnosis. Therefore, we believe that no severe symptoms were induced in all cases. However, most patients showed positive reactions at the first loading dose (1/50 full dose). In only patients with a previous history of severe reactions, a lower starting dose of 0.06 g protein/ kg body weight is recommended in international consensus guidelines [1]. However, a study in Canada [13] modified the OFC for patients with acute FPIES, starting from a tiny dose of 1/100 full dose. The initial dose and interval of each loading in the Canadian study were lower and longer than the international guidelines and our protocol. The modification of the current OFC protocol, based on international consensus guidelines [1], might be required for patients with severe acute FPIES, with a first loading dose of 1/100 full dose. In a study in Canada, the OFC protocol has the disadvantage of a long time to full dose. Based on the severity of the patient and the feasibility of the protocol, further consideration is needed to update the FPIES remission OFC protocol. In this study, IVF was administered in 50% of positive cases. As recommended by the FPIES international guidelines [1], it is advisable to have intravenous access available before starting OFC in children. In addition, some cases developed reactions exceeding 4 h of the diagnostic criteria of the guidelines. Even after 4 h from the start of OFC, a careful follow-up is necessary, considering the possibility of acute FPIES symptoms.

A single food In most children causes FPIES [14]. In the USA, milk and soy were common causal foods in children [15]. In a large Australian cohort (n = 230), rice was the most common food trigger (45%), followed by milk (33%) and egg (12%) [16]. Fish was a common trigger in infants from Italy and Spain [17,18]. Feeding practice, age of introduction of a specific food, and genetic predisposition might underpin the geographic differences in patients with FPIES. Although patients with IgE-mediated food allergy caused by egg yolk are rare, reports of egg yolk FPIES have been increasing in recent years [3,19,20].

The strength of this study was that the assessment of acute FPIES was performed using standardized protocol based on the international FPIES guidelines. This study has several limitations. First, we reported from a single center. Second, this was a retrospective study. Third, the relationship between causative food and OFC outcomes could not be evaluated due to the small sample size. Fourth, we had several missing data, given the retrospective design of this study. However, a multicenter prospective cohort study is now being planned for a large number of patients with acute FPIES. Further study is expected to overcome these limitations.

## 5. Conclusions

Twenty-three patients with acute FPIES who underwent OFC for remission evaluation were reviewed. The remission of acute FPIES was observed in 65.2% of patients, and the median duration between the onset and OFC was 14 months (IQR, 8–24). However, vomiting episodes occurred with 1/50 full dose on the first day among 75% of positive patients. This study suggested that our OFC protocol for acute FPIES was safely performed without inducing severe symptoms; however, it might be safer for some patients to start a minimal loading dose.

## Figures and Tables

**Table 1:** Diagnostic criteria for acute FPIES of international consensus guidelines [1].

Major Criterion:
Vomiting in the 1 to 4 h period following the ingestion of the suspect food and absence of classic IgE-mediated allergic skin or respiratory symptoms.
**Minor criteria:**
1. A second (or more) episode of repetitive vomiting after eating the same suspect food; 2. Repetitive vomiting episode 1–4 h after eating a different food; 3. Extreme lethargy with any suspected reaction; 4. Marked pallor with any suspected reaction; 5. Need for emergency department visit with any suspected reaction; 6. Need for intravenous fluid support with any suspected reaction; 7. Diarrhea in 24 h (usually 5–10 h); 8. Hypotension; 9. Hypothermia.

The diagnosis of FPIES requires that a patient meets the major criterion and >3 minor criteria. If only a single episode has occurred, a diagnostic OFC should be strongly considered to confirm the diagnosis, especially because viral gastroenteritis is extremely common in this age group. Furthermore, although not a criterion for diagnosis, it is important to recognize that acute FPIES reactions will typically completely resolve over a matter of hours, compared with the usual several-day time course of gastroenteritis. The patient should be asymptomatic and grow normally when the offending food is eliminated from the diet.

**Table 2:** Clinical characteristics of patients with acute FPIES and risk factor analysis with OFC outcomes.

	All (n = 23)	OFC-Positive (n = 8)	OFC-Negative (n = 15)	*p*-Value
**Male, no. (%)**	11 (47.8)	5 (62.5)	6 (40.0)	0.555 *
**Age at onset of initial reactions (month), median (IQR †), range**	7.0 (6.25–8.0), 0–15	8.0 (6.0–8.25), 0–14	7.0 (6.0–7.5), 0–15	0.324 **
**Age at diagnosis (month), median (IQR †)**	8.0 (6.25–11.5)	11.5 (7.25–17.8)	7.0 (6.0–9.0)	0.217 **
**Causative food, no. (%)**				
**Soy**	8 (34.8)	1 (12.5)	7 (46.7)	0.136 *
**Egg yolk**	5 (21.7)	4 (50.0)	1 (6.7)	
**Milk**	3 (13.0)	2 (25.0)	1 (6.7)	
**Wheat**	3 (13.0)	1 (12.5)	2 (13.3)	
**Egg white**	2 (8.7)	0 (0.0)	2 (13.3)	
**Rice**	1 (4.4)	0 (0.0)	1 (6.7)	
**Fish**	1 (4.4)	0 (0.0)	1 (6.7)	
**Skin prick test for trigger foods positivity, no. (%) §§**	0 (0.0)	0 (0.0)	0 (0.0)	-
**Specific IgE antibody positivity, no. (%) ‡**	3 (13.0)	1 (12.5)	2 (13.3)	0.550 *
**Additional allergic conditions, no. (%)**				
**Atopic dermatitis**	9 (39.1)	5 (62.5)	4 (26.7)	0.221 *
**Asthma**	1 (4.4)	0 (0.0)	1 (6.7)	0.742 *
**Allergic rhinitis**	1 (4.4)	1 (12.5)	0 (0.0)	0.742 *
**IgE-food allergy**	2 (8.7)	2 (25.0)	0 (0.0)	0.213 *
**Birth history of cesarean section, no. (%)**	11 (47.8)	5 (62.5)	6 (40.0)	0.551 *
**Birth weight (g) §**	2963 ± 429	2956 ± 575	2996 ± 413	0.560 **
**Breastfeeding at onset, no. (%)**	17 (73.9)	6 (75.0)	11 (73.3)	0.681 *
**Family allergic disease history, no. (%)**	15 (65.2)	5 (62.5)	10 (66.7)	0.795 *
**Bodyweight at OFC (kg) ‡**	11.1 ± 2.7	11.3 ± 2.95	10.8 ± 2.54	0.557 ***
**White blood cell (/μL) §**	9631 ± 3290	8076 ± 1670	10,306 ± 3656	0.158 ***
**Neutrophil (%) §**	31.8 ± 11.8	36.9 ± 9.97	29.5 ± 12.2	0.086 ***
**Eosinophil (%) §**	2.80 ± 2.32	3.06 ± 2.82	2.69 ± 2.07	0.574 ***
**CRP (mg/dL) §**	2.80 ± 2.32	1.25 ± 2.09	0.30 ± 0.91	0.297 ***
**Total IgE (IU/mL) §**	80.9 ± 170	52.6 ± 62.3	94.8 ± 201	0.519 ***
**Specific IgE antibody (U_A_/mL) §**	0.170 ± 0.194	0.14 ± 0.01	0.18 ± 0.23	0.543 ***
**TARC (pg/mL) §**	718 ± 581	961 ± 857	589 ± 335	0.314 ***
**Age in months OFC performed, median (IQR †), range**	21.0 (15.3–32.0), 6–56	23.5 (15.5–33.3), 7–56	19.0 (14.5–31.5), 6–45	0.722 **
**Duration between onset and OFC (months)**	14 (8–24)	14 (7.75–20.75)	13 (8.5–23.5)	0.897 **

* *p*-value was calculated using Fisher’s exact test. ** *p*-value was calculated using Mann–Whitney U-test. *** *p*-value was calculated using Student’s *t*-test. † 25% to 75% interquartile range. ‡ IgE positivity was defined as detectable serum food-specific IgE (>0.35 kUA/L). § Mean ± SD. §§ evaluated number was 22 (one patient was missing). *FPIES*, food protein-induced enterocolitis syndrome. *OFC*, oral food challenge.

**Table 3:** Clinical characteristics of subjects who had positive reactions in OFC.

Patient No.	1	2	3	4	5	6	7	8
**Sex**	Female	Male	Male	Male	Male	Female	Female	Male
**Food**	Milk	Milk	Egg yolk	Egg yolk	Egg yolk	Wheat	Egg yolk	Soy
**Initial diagnosis**	Acute FPIES	Acute FPIES or FPIAP	Acute FPIES	Acute FPIES	Acute FPIES	Acute FPIES	Acute FPIES	Acute FPIES
**Age at onset (m)**	0	0	9	8	14	8	8	8
**Age at diagnosis(m)**	1	0	16	13	26	10	15	8
**Age at OFC (m)**	7	14	56	25	32	22	37	16
**First reaction of acute FPIES (times)**	Vomiting (2)	Bloody stool Vomiting (2)	Vomiting (3) Diarrhea (2)	Vomiting (10)	Vomiting (frequent)	Vomiting (5) Diarrhea (1)	Vomiting (2) Lethargy	Vomiting (4) Diarrhea (1)
**Time (h)**	3–5	Unknown	2	3	2	5	2	4
**Severity in the first reaction**	Mild	Mild	Moderate	Moderate	Moderate	Moderate	Moderate	Moderate
**OFC reaction (times)**	Vomiting (4)	Vomiting (3)	Vomiting (2) Diarrhea (1) Abdominal pain	Vomiting (4)	Vomiting (3) Abdominal pain Lethargy	Vomiting (3)	Vomiting (2) Lethargy	Vomiting (1) Lethargy
**Time (h)**	2.5	8	2	2.5	2	2	3	5
**Day (day)**	1	2	1	1	1	4	1	1
**Food protein of body weight at the reaction (g/kg)**	0.008	0.03	0.006	0.007	0.006	0.3	0.007	0.006
**Severity in OFC**	Moderate	Moderate	Mild	Moderate	Moderate	Moderate	Moderate	Mild
**Treatment**	Intravenous normal saline bolus	Intravenous normal saline bolus	None	Intravenous normal saline bolus and corticosteroids	None	Intravenous normal saline bolus and corticosteroids	Intravenous normal saline bolus	None
**Confirmatory diagnosis by OFC**	Acute FPIES	Unclassifiable †	Acute FPIES	Acute FPIES	Acute FPIES	Acute FPIES	Acute FPIES	Acute FPIES

† likely acute FPIES; however, the first vomiting episode appeared for more than 4 h. *FPIES*, food protein-induced enterocolitis syndrome. *FPIAP*, food protein-induced allergic proctocolitis. *OFC*, oral food challenge.

## Data Availability

The data supporting this study’s findings are available from the corresponding author, K.N., upon reasonable request.

## Supplementary Materials

The following supporting information can be downloaded at: https://www.mdpi.com/article/10.3390/nu14194158/s1, Table S1: OFC Protocol in our hospital setting and the challenge dose.
