# Supplementary material for: Remission of Acute Food Protein-Induced Enterocolitis Syndrome Confirmed by Oral Food Challenges in Japan
Source: Nutrients. 2022 Oct 7;14(19):4158. doi: 10.3390/nu14194158 (PMC9570973; doi:10.3390/nu14194158)
Supplement: Supplementary file 1 [file nutrients-14-04158-s001.zip › nutrients-1932763-supplementary.pdf]

Supplementary Table S1 OFC Protocol in our hospital setting and the challenge dose.

Mon (Day 0)	Tue (Day 1)	Wed (Day 2)	Thu (Day 3)	Fri (Day 4)	Sat (Day 5)
Admission Skin prick test	1/50 serving size	1/10 serving size	1/2 serving size	Full serving size	Discharge

The target challenge dose was set based on age (change according to severity)
OFC, oral food challenge. If the symptomatic episode was severe, the target protein loading dose was set at a low dose (0.06 g/kg). If the symptomatic episode was mild to moderate, the target protein loading dose was set at a standard dose (0.3 g/kg).
The target dose was started at 1/50th, and it increased in the following order: 1/50th, 1/10th, 1/2, and then a full dose. The full challenge dose is not to exceed 3 g protein or 10 g whole food.

（ex）loading dose for a 10-kg infant

**Food**	**FPIES severity**	**Loading food dose (protein content in foods)**			
		**(Day 1)** 1/50	**(Day 2)** 1/10	**(Day 3)** 1/2	**(Day 4) Full**
**Egg Yolk**	Severe	0.06g (0.0012g/kg)	0.3g (0.006g/kg)	1.5g (0.03g/kg)	3g (0.06g/kg)
	Mild to moderate	0.3g (0.006g/kg)	1.6g (0.03g/kg)	8g (0.15g/kg)	16g (0.3g/kg)
**Egg White**	Severe	0.12g (0.0012g/kg)	0.6g (0.006g/kg)	3g (0.03g/kg)	6g (0.06g/kg)
	Mild to moderate	0.6g (0.006g/kg)	3g (0.03g/kg)	15g (0.15g/kg)	30g (0.3g/kg)
**Lactose-free milk**	Severe	0.7ml (0.0012g/kg)	3.5ml (0.006g/kg)	17ml (0.03g/kg)	35ml (0.06g/kg)
	Mild to moderate	3.5ml (0.006g/kg)	18ml (0.03g/kg)	88ml (0.15g/kg)	177ml (0.3g/kg)
**Soy (Tohu)**	Severe	0.2g (0.0012g/kg)	1.2g (0.006g/kg)	6g (0.03g/kg)	12g (0.06g/kg)
	Mild to moderate	1.2g (0.006g/kg)	6g (0.03g/kg)	30g (0.15g/kg)	60g (0.3g/kg)
**Rice**	Severe	0.5g (0.0012g/kg)	2.4g (0.006g/kg)	12g (0.03g/kg)	24g (0.06g/kg)
	Mild to moderate	2.4g (0.006g/kg)	12g (0.03g/kg)	60g (0.15g/kg)	120g (0.3g/kg)
